# The Impact of Large-Scale Expansion on the Functional Properties of Mesenchymal Stem Cells

**DOI:** 10.1007/s12015-026-11068-x

**Published:** 2026-02-06

**Authors:** Guadalupe Antonio-Ríos, Rosa María Ribas-Aparicio, Gerardo Leyva-Gómez, Gloria Soldevila, Karla Alejandra Espinoza Dueñas, Cynthia Georgina Trejo-Iriarte, Maykel González-Torres

**Affiliations:** 1 Laboratorios de Producción y Control de Biológico, Biotecnología Molecular y Farmacéutica, Departamento de Microbiología, Escuela Nacional de Ciencias Biológicas, Instituto Politécnico Nacional, Ciudad de México, 11340 México; 2 Departamento de Farmacia, Facultad de Química, Universidad Nacional Autónoma de México, Ciudad de México, México; 3 Departamento de Inmunología y Laboratorio Nacional de Citometría de Flujo, Instituto de Investigaciones Biomédicas, Universidad Nacional Autónoma de México, Circuito Escolar s/n, Ciudad Universitaria, Colonia Copilco, Delegación Coyoacán, Ciudad de México, 04510 Mexico; 4 Centro de Graduados e Investigación, Instituto Tecnológico de Tijuana, Blvd. Limón Padilla S/N, Mesa de Otay, Apartado Postal 1166, Tijuana, BC 22000 México; 5 Programa de Cirugía Dental, Laboratorio de Investigación en Odontología Almaraz, Facultad de Estudios Superiores Iztacala, UNAM, Cuautitlán Izcalli, Estado de México 54714 México; 6 Departamento de Ciencias Químicas, Facultad de Estudios Superiores Cuautitlán, Universidad Nacional Autónoma de México, Cuautitlán Izcalli, Estado de México México

**Keywords:** Adipose-derived stem cells, Large-scale culture expansion, Mesenchymal stem cells, Regenerative medicine, Therapeutic potential, Cell functionality

## Abstract

Mesenchymal stem cells (MSCs) are highly valuable for their potential in cell therapy and tissue engineering because of their self-renewal, multilineage differentiation, and immunomodulatory capabilities. Adipose-derived mesenchymal stem cells (AD-MSCs) are advantageous in regenerative medicine because of their accessibility and ease of isolation. However, the clinical application of MSCs faces challenges related to large-scale culture (LSC) expansion, which is required to generate enough cells for transplantation but also decreases their therapeutic properties. This review assesses the impact of LSC on MSC functionality, differentiation potential, and immunomodulatory properties, and identifies key factors, such as metabolic shifts, genetic instability, and altered secretory profiles, that can compromise their therapeutic potential. We explored how prolonged in vitro passaging decreases MSC functionality and increases the risk of genetic alterations. In addition, strategies to preserve the efficacy of MSCs during scaling are discussed. A comprehensive literature review was conducted using PubMed, focusing on in vitro and in vivo studies that evaluated the effects of LSC on MSCs. These findings provide insights into optimizing culture protocols to maintain the clinical efficacy of AD-MSCs in regenerative therapies, addressing the critical need to balance large-scale expansion and functional integrity.

**Graphical Abstract:**

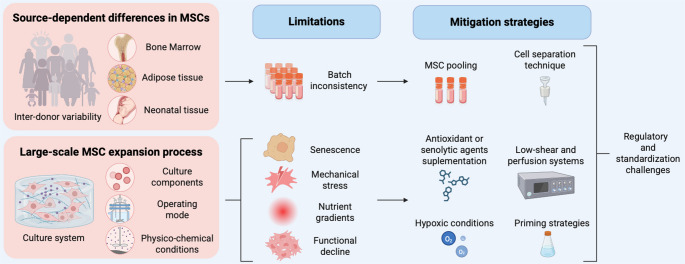

## Introduction

 Mesenchymal stem cells (MSCs) are adult stem cells characterized by their regenerative ability and multilineage differentiation potential into specialized cell types, such as osteoblasts, chondrocytes, and adipocytes [1]. MSCs are the most commonly tested adult stem cells in experimental cell therapy because of their ability to undergo multilineage differentiation for damaged tissue replacement and their capacity to modulate adaptive and innate immune responses [2]. Additionally, numerous studies have highlighted the antiapoptotic, antioxidative, proangiogenic, regenerative, and antifibrotic properties of MSCs. These effects are mediated either through direct cell–cell contact or paracrine signaling mechanisms [3]. However, the transition of MSC-based therapies from bench to bedside, requires the production of clinically relevant cell numbers, which poses substantial challenges for large-scale manufacturing.

A central obstacle in MSC biomanufacturing is the balance between scalability and preservation of functional attributes [4]. Clinical applications require the expansion of MSCs without compromising their therapeutic effectiveness. These expansion processes involve the maintenance of the phenotypic and functional properties of cells [5]. However, as MSCs undergo repeated passaging during in vitro expansion, they are exposed to various stressors that may reduce their functionality. These challenges include metabolic changes, alterations in secretory profiles, and impaired immunomodulation [2]. Prolonged culture also increases the risk of genetic instability and reduces cell viability, which could compromise the clinical effectiveness of MSC-based therapy [6]. Therefore, it is critical to understand how large-scale culture affects MSC characteristics and to develop strategies to mitigate these risks.

This narrative review provides an integrated analysis of the biological and biotechnological factors influencing MSC quality during large-scale expansion. We synthesize recent advances in culture platforms, including two-dimensional (2D) and three-dimensional (3D) systems, microcarrier technologies, and dynamic bioreactors, and critically examine their impact on immunophenotype, differentiation potential, senescence, and potency. In addition, we discuss the current quality control measures and highlight emerging tools, such as deep learning–based senescence detection and omics-based release assays. The article concludes by outlining the current technical bottlenecks and proposing future strategies for improving MSC biomanufacturing, with the goal of increasing safety, consistency, and therapeutic efficacy.

This review introduces a unified conceptual framework integrating replicative senescence, functional drift, and bioprocess-induced stress to explain how large-scale expansion compromises MSC quality. By synthesizing Population Doubling Level (PDL) thresholds, bioreactor-specific effects, and emerging GMP potency assays, this article provides a structured and novel perspective on how scalability challenges can be mitigated in clinical manufacturing.

## Large-Scale Expansion Techniques for MSCs

### Overview of Clinical-Scale Culture Systems

The expansion of MSCs to clinical-scale volumes requires specialized culture systems with the capacity to produce large quantities of cells while maintaining their functions. Large-scale expansion can be performed using different strategies, with advantages and disadvantages that vary depending on the scale.

### 2D Planar Expansion Systems

2D culture systems, in which cells grow as a monolayer and attach to a plastic surface, are commonly used for MSCs expansion. These systems include tissue culture-treated flasks and multilayer vessels. Multilayer vessels are redesigned culture flasks consisting of multiple layers of cell culture-treated surfaces that offer ample space for cell growth. These static systems are more compact than traditional cultured T-75 or T-175 flasks and save considerable space in the incubators. This group includes the HYPERFlask, CellSTACK, and Cell Factory systems, which vary in surface area from 1272 cm² to 3180 cm² [7]. Although 2D culture systems are commonly used owing to their simplicity and cost-effectiveness, planar systems have disadvantages, as they require excessive manipulation, pipetting, and dispensing, which increases the risk of contamination and limits the control and monitoring of the culture conditions [8]. Despite these limitations, some studies have shown that early passage MScs expanded in 2D culture systems can maintain their immunophenotype and trilineage differentiation capacity [9, 10]. As the growth environment of 2D cultures is not completely consistent with that of cells in vivo, 3D systems are alternatives that provide cells with a culture environment that more closely resembles the in vivo milieu [11]. Although widely used, 2D systems provide limited environmental control and are less suitable for large-scale biomanufacturing, which has motivated the adoption of 3D and dynamic bioreactor platforms.

### 3D and Dynamic Bioreactor Systems

In contrast to planar systems, 3D and dynamic bioreactors provide improved scalability, environmental standardization, and process control, which are suitable for clinical-scale MSC production. 3D culture has been proposed as an effective strategy for large-scale cell production and improved bioactivity [12, 13]. 3D models can be divided into suspension cultures on non-adherent plates, cultures in concentrated medium or gel-like substances, and cultures on a scaffold [14]. Spinner flasks are basic models for 3D culture with mechanical agitation for the distribution of nutrients [15] and conditions, such as oxygen, pH, glucose, lactate, and microcarriers, where cells grow. This 3D dynamic culture system, created by mixing MSCs with modified polyethylene glycol (PEG) and hyaluronic acid, has been shown to enhance protein secretion and therapeutic efficacy in large, deep burns. Compared with traditional 2D methods, this system results in enhanced cell proliferation and stable culture conditions [12]. Nevertheless, a dynamic culture system creates shear stress on cells because it involves mechanical agitation of the culture medium or vessel to allow more efficient nutrient transfer [7]. In addition, spinner and multilayer flasks involve excessive manipulation, pipetting, and dispensing, which increases the risk of contamination and limits the control and monitoring of culture conditions. In contrast, closed 3D systems are equipped with technologies for monitoring and controlling the parameters of the culture environment, such as temperature, pH, nutrients, metabolites, and gases [8].

Different closed bioreactor systems have been tested for large-scale in vitro amplification of MSCs, with stirred tanks, rocking, rotating beds, hollow fibers, and fixed beds being the most widely used systems [4]. Stirred tank bioreactors consist of vessels with internal impellers that create a dynamic environment for suspension cultures, where cells grow as aggregates or in microcarriers. Rocking bioreactor systems have been designed to induce waves in cell culture media to suspend cells and grow them in suspension as 3D aggregates or in microcarriers. Rotating bed bioreactors comprise a cylindrical culture vessel with an integrated rotating slide that supports a scaffold for cell proliferation. HFPE bioreactors are composed of many capillaries and semipermeable hollow fibers located in a tubular housing, where MSCs are cultured in the intracapillary space of the fibers to attach and grow in a 3D configuration. Fixed-bed bioreactors consist of an immobilized scaffold in a disposable sterile bottle, on which adherent cells attach and grow, forming a dense 3D environment [4]. All these bioreactor systems can be operated in different modes to modify feeding strategies, including batch, repeated batch, fed-batch, and perfusion [8].

Among bioreactor systems, microcarrier-based stirred tanks improve cell proliferation and maintain a more youthful state compared to cells cultured under planar conditions [16]. Microcarriers function as good scaffolds for MSCs and allow them to proliferate while maintaining an undifferentiated state, thus contributing to large-scale MSC culture [17].

Although stirred tank bioreactors are the most widely used platform for large-scale MSC manufacturing, they still face challenges, such as the generation of cell microcarrier clumps and the implementation of the most suitable cell harvesting process [4]. Therefore, microcarrier surface modifications are necessary to improve cell attachment and harvesting techniques [18].

Among the different bioprocessing strategies for MSC expansion, multilayer flasks, spinner flasks, and bioreactors are the most commonly used methods. Compared with spinner flasks and roller bottles, multilayer flasks and bioreactors can achieve higher expansion ratios [7]. Nonetheless, varying culture conditions can affect the number and quality of the cells.

### Optimization of Culture Conditions

**Fig. 1:**
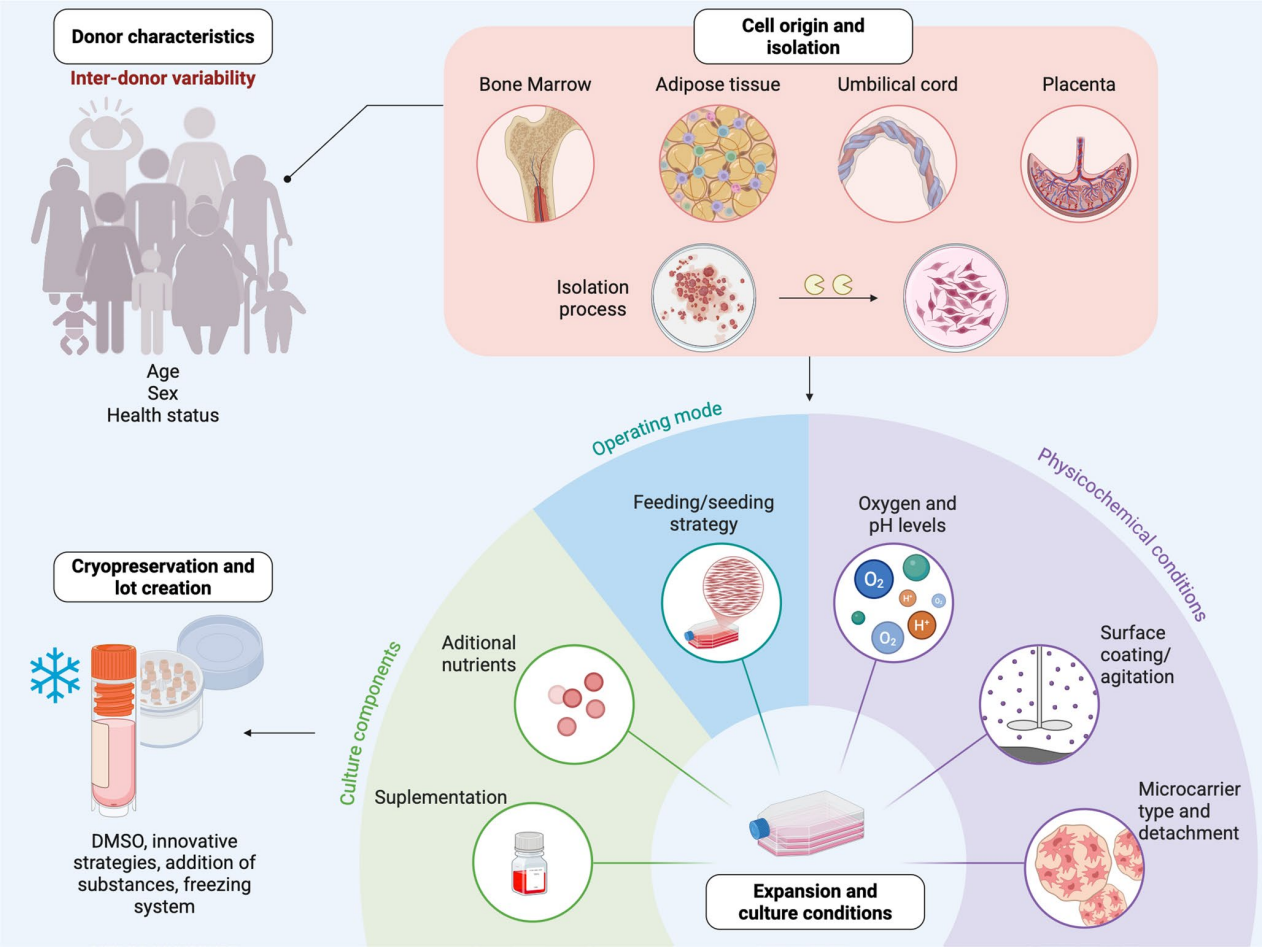
Variations in bioprocessing strategies for MSC expansion can modify cell behavior. The figure shows four critical conditions during MSC culture that impact their functional and immunomodulatory properties (Created with BioRender.com)

Several factors can influence MSC behavior during clinical application, making the optimization of culture conditions essential to ensure the success of these therapies. These factors include the inherent properties of MSCs, which are related not only to their donor characteristics and cell origin, but also to their isolation process, expansion and culture conditions, and cryopreservation methods (Fig. 1).

### Donor Characteristics Influence MSCs Properties

MSCs possess inherent properties related to donor characteristics, such as age, sex, and health status, which should be considered when selecting ideal donors for MSC therapy. Some maternal and fetal factors influence the yield of Wharton’s Jelly derived MSCs (WJ-MSCs). WJ-MSC output was positively associated with younger maternal age, greater gestational age, and higher birth weight. In contrast, factors such as maternal parity, neonatal sex, and head circumference do not affect WJ-MSC behavior [19]. In addition, the self-renewal ability of placenta-derived MSCs (PD-MSCs) is greater in donors aged 26–30 years than in younger or older donors. Interestingly, the increase in the proliferative capacity of PD-MSCs was correlated with increased telomere shortening, suggesting that shorter telomere lengths may be related to cellular division rather than aging [20]. In addition to telomere shortening, decreased proliferation and a decline in adipogenic capacity are related to aging because of the reduction in cell nucleus size in patients, as shown in AD-MSCs [21]. However, the effects of age on functional and paracrine function remain unclear [22].

Moreover, the potency and functionality of both female and male MSCs have been studied previously. Adipose stem cells display sexual dimorphism in genes involved in estrogen signaling, homeobox transcription factor expression, and the renin-angiotensin-aldosterone system, which can be attributed to the effects of sex hormones [23]. A study on WJ-MSCs revealed that OCT4 and AND-methyltransferase 1 (DNMT1) gene expression was greater in MSCs isolated from males than in those isolated from females. OCT4 exerts tight control over pluripotency regulator expression and protects undifferentiated embryonic stem cells; thus, it has a specific relationship with stemness genes, epigenetic modulators, and sex differences [24]. This finding is consistent with that of another study in which umbilical cord MSCs (UC-MSCs) were isolated from heterosexual twins. One study reported that male fetal UC-MSCs have greater proliferation and adipogenic ability than female fetal UC-MSCs and that the expression levels of NANOG, OCT4, TERT, and SOX2 are significantly different between males and females. Similarly, male MSCs express higher levels of inflammatory cytokines in response to lipopolysaccharide-induced inflammation [25]. In contrast, female AD-MSCs exhibit more potent immune modulation in vitro than male AD-MSCs [26]. This topic is described in detail in the Impact of Large-Scale Expansion on Immunosuppressive Properties section of this manuscript. In addition, Maged, Abdelsamed [27] reviewed the impact of donor sex on MSC behavior.

Furthermore, the health status of the donor should be considered when selecting ideal MSCs donors. For example, obesity negatively affects the senescence and differentiation potential of MSCs, particularly their mitochondrial function and epigenetic profiles, which are crucial for their reparative abilities [28]. In vitro assays and AD-MSCs from high-fat diet-fed murine models revealed a senescent phenotype, altered cell cycle distribution, and loss of stemness due to a hostile environment with oxidative stress in the adipose tissue [29].

### The Cell Origin and Isolation Process Influence MSCs Properties

The origin of the cell affects MSCs behavior. Compared with UC-MSCs, AD-MSCs have a shorter doubling time and a greater population doubling level, which improves the cellular yield in less time. This renders AD-MSCs a viable option for clinical situations that require immediate single-dose administration of cells. However, UC-MSCs retain their self-renewal ability at higher passage numbers than AD-MSCs [30]. Therefore, selecting the most appropriate tissue source for MSCs is necessary to maximize their therapeutic potential and ensure optimal outcomes in regenerative medicine.

Isolation protocols are also important because enzymatic digestion combined with mechanical distortion improves AD-MSC recovery, in contrast to enzymatic digestion alone [31]. Additionally, compared with the explant method, enzymatic digestion methods avoid faster outgrowth [9].

### Population Doubling Level (PDL) Limits and Functional Decline

PDL is a critical parameter that influences MSC quality during large-scale expansion, as it reflects the cumulative number of mitotic events and is closely associated with replicative senescence, telomere erosion, and functional decline of MSCs. PDL limits vary depending on the tissue source, donor characteristics, and culture medium composition of the cells. MSCs derived from adipose tissue typically reach higher PDLs in shorter time frames but also show an earlier onset of functional drift, including reduced clonogenicity and alterations in metabolic and secretory profiles. In contrast, umbilical cord–derived MSCs (UC-MSCs) maintain self-renewal capacity in more population doublings, resulting in slower telomere shortening and delayed senescence-associated gene expression, although their differentiation potential and immunomodulatory properties eventually deteriorate at higher PDLs. Donor age is another determinant of PDL thresholds: placenta-derived MSCs from donors aged 26–30 years retain a greater proliferative capacity before senescence, whereas MSCs from older donors exhibit accelerated telomere attrition and reduced adipogenic capacity, leading to an earlier functional decline. The composition of the culture medium further modulates the decay dependent on PDLs; serum-free and xeno-free formulations tend to prolong the proliferative lifespan, preserve genomic stability, and delay senescence markers compared with fetal bovine serum–containing media. Supplements such as nucleosides or niacinamide have been shown to support higher PDLs without compromising the functionality of MSCs. Together, understanding the PDL-associated thresholds in MSC sources and culture conditions is essential for defining passage limits, optimizing manufacturing workflows, and mitigating functional deterioration during large-scale expansion of MSCs.

### Expansion and Culture Conditions Influence MSC Properties

When MSCs are subsequently isolated, the culture conditions, such as the culture medium components and physicochemical conditions, can affect MSC behavior. The composition of the culture medium alters the biological attributes of MSCs, such as clonogenicity, proliferation, differentiation propensity, and immunomodulatory capacity [32]. Regular monitoring of media composition is crucial for negatively affecting cell growth and function [33]. The media can include components such as nucleosides, which enable faster cell proliferation and maintain MSC functionality during large-scale culture [34]. Additionally, the addition of niacinamide can enhance MSC growth while supporting their immunomodulatory phenotype, which is linked to their differentiation potential [35].

If the obtained MSCs are to be used as therapeutic components, they must comply with regulatory standards to ensure their safety and efficacy in clinical applications, necessitating rigorous specifications during manufacturing. Clinical-grade MSC production typically requires adherence to good manufacturing practices (GMPs), which necessitate the avoidance of fetal bovine serum (FBS). These xeno-free or serum-free alternatives modify or enhance the reproducibility and quality of the final cell product, ultimately leading to improved therapeutic outcomes [36].

Human platelet lysate (hPL) is an alternative to FBS for clinical-grade MSCs, as it reduces the risk of immunoreaction. Autologous or allogeneic human serum can be used to reduce the degree of immunogenicity. However, supply limitations and variability pose significant challenges to their use in clinical practice. Standardized MCS GMP medium, composed of serum- and xenogenic-free media, is an alternative to hPL for MSC expansion [37]. Commercial serum- and xenogenic-free media are specifically designed for MSC culture under GMP conditions without the use of human-derived components and are suitable for large-scale expansion [38]. MCS-Brew GMP medium is manufactured in bags suitable for clinical-scale expansion and does not require precoating of culture surfaces or the addition of blood derivatives during the isolation step [37]. Compared with serum-containing media, serum-free media increase proliferation, reduce the senescence rate, and maintain a normal karyotype [36].

Physical factors also affect MSC behavior, such as hypoxic conditions that increase proliferation and immunoregulatory function while maintaining a normal karyotype and inhibiting senescence [39]. Moreover, changes in nanotopography help maintain the naïve multipotent phenotype of MSCs and change cell adhesion and intracellular tension during large in vitro cultures by promoting oxidative glycolysis. These findings emphasize the potential of glycolysis-driven metabolites [35]. Indeed, the use of hPL as a coating material can improve AD-MSCs cultures by increasing the cell adhesion rate, shortening population doubling times, stimulating cell growth, and downregulating senescence-associated genes, such as p16, p21, and p53 [40]. However, the use of hPL requires careful management and quality control to ensure its safety and efficacy in clinical applications because of batch-to-batch variability, risk of pathogen transmission, and quality control challenges [41, 42].

During the bioprocessing strategy for MSC expansion, specific expansion systems and operation mode selection are important factors to consider because they can significantly affect cell yield and functionality. Compared with cells expanded through planar culture, UC-MSCs expanded on microcarriers presented a lower degree of cellular aging, increased proliferation capacity, reduced number of cells in the cell cycle retardation period, and altered expression of genes associated with the cytoskeleton and extracellular matrix [16]. Additionally, gellan gum polymer-based 3D cultures increase the expression of stemness markers and immunomodulatory molecules compared to 2D cultures [43]. Additionally, 3D hypoxic system culture enhances MSC production and cellular properties, such as angiogenesis, immunosuppression, and anti-aging effects [44].

The operation mode is an important factor for optimizing the nutrient supply (such as glucose and glutamine) and waste management (such as lactate and ammonia) to increase MSC proliferation, which can be moderated through a fed-batch mode with medium replacement [15]. Furthermore, the combination of a microcarrier-based dynamic culture system with a fed-batch mode and medium replacement improves nutrient supplementation and reduces the amount of medium used for cell culture, resulting in a greater than 20-fold increase in the number of cells [15]. Finally, when MSCs are cryopreserved, the preservation and defrosting procedures can modify their characteristics [45].

### Impact of Large-Scale Expansion on Differentiation Potential

In addition to maintaining the functional properties of MSCs after expansion, MSCs must maintain their fundamental characteristics, such as surface markers and differentiation potential. As differentiation potential is a critical feature of MSCs in regenerative medicine, understanding how clinical-scale culture affects this property is crucial for optimizing therapeutic results.

Regarding variations between tissue sources, the expression of pluripotency factors such as NANOG, OCT4, and SOX2 did not differ significantly. MSCs derived from different tissue sources retain the capacity to express pluripotent genes [30]. Although MSCs can differentiate into adipocytes, osteocytes, and chondrocytes independently of their tissue of origin, there is a preference for certain lineages depending on their source of origin. For example, dental pulp MSCs (DP-MSCs) have a better osteogenic differentiation potential than AD-MSCs. Moreover, osteogenic differentiation can be enhanced by the activation of BMP signaling with BMP-2, which increases the expression of activin receptor-like kinase-3 and − 6. However, DP-MSCs that underwent replicative senescence (passage ≥ 10) exhibited reduced osteogenic differentiation, and treatment with BMP-2 did not enhance this effect. In comparison, AD-MSCs retain a similar osteogenic differentiation potential, especially at early and late passages [46]. In addition, UC-MSCs have a lower adipogenic potential and require more time for osteogenic differentiation than AD-MSCs and BM-MSCs [38].

Donor characteristics also influence MSC differentiation capacity. For example, multipotency and differentiation markers exhibit increased expression in PD-MSCs from mothers aged 22–35 years, with a sevenfold increase in adipogenesis [20]. Donor sex also influences differentiation capacity; for example, male fetal UC-MSCs have greater adipogenic ability than female fetal UC-MSCs, and twins show significant differences in the expression levels of NANOG, OCT4, TERT, and SOX2.

Therefore, an increasing number of studies have examined the effects of large-scale cultures on MSC differentiation. Some studies have indicated that the ability of MSCs to differentiate into lipid, osteogenic, and cartilage lineages is not altered by the culture method used, similar to the utilization of MC bioreactors for UC-MSCs [15, 16] and BM-MSCs [33]. This differentiation potential is not altered or changed in culture media [37] or in serum-containing and serum-free media [33]. In addition to MCs, other types of nanotopography can be used in surface cultures to retain the naïve multipotent phenotype for prolonged periods, which is essential for their differentiation capabilities [35].

Table 1 summarizes recent studies that evaluated the effects of different strategies for large-scale expansion on the differentiation capacity of MSCs. Despite methodological heterogeneity, common trends have emerged regarding the preservation of multipotency under optimized conditions. During MSC scale-up in bioreactor systems, agitation dynamics affect differentiation potential. Intermittent stirring during the early adhesion phase enhances cell attachment while preserving trilineage differentiation potential [47, 48]. However, excessive agitation rates may compromise stemness and induce spontaneous differentiation, as observed in UC-MSCs [49]. Other biophysical and chemical culture conditions also influence lineage commitment. For example, hypoxic conditions promote osteogenic differentiation while reducing adipogenic potential, in contrast to 2D expanded MSCs [44]. Pulsed electromagnetic fields also increase the expression of osteogenic genes [50, 51]. The passage number is a critical determinant of the differentiation potential. MSCs derived from Wharton’s Jelly retain their trilineage differentiation potential up to passage five, although their osteogenic capacity begins to decrease thereafter [9]. The pluripotency of stirred-tank or multilayer systems is preserved in serum-containing or xeno/serum-free media [10, 52]. Innovative approaches, such as 3D spheroid culture and the use of dissolvable microcarriers, effectively support multilineage differentiation. Additionally, these strategies enhance immunomodulatory features and allow for the dual production of MSCs and extracellular vesicles [53, 54]. In general, when large-scale expansion is appropriately optimized, the differentiation potential of MSCs preserves. For example, critical variables, such as agitation rate, oxygen level, passage number, and bioreactor configuration, must be controlled to preserve their functional properties.

**Table 1:** Key factors in the large-scale expansion of MSCs and their impact on differentiation potential [9, 10, 44, 47–56]

Cell source	Expansion method	Factor	Effect on differentiation potential	Extra notes	Ref.
Warthon’s Jelly	Spinner flask	Seeding density, agitation and culture feed regime	Maintains trilineage differentiation potential	Intermittent stirring and reduced supplementation during the attachment phase improve cell adhesion	[48]
Warthon’s Jelly	Stirred tank bioreactor	Serum containing and xeno and serum-free media	Trilineage differentiation potential is comparable to 2D culture	Serum-free media in 2D has better initial cell attachment	[52]
Warthon’s Jelly	Multilayer flask	Passage number	Passage 1–5 demonstrated trilineage differentiation potential	Osteogenic differentiation capacity decline at passage 5	[9]
Umbilical cord	Stirred tank bioreactor	Hypoxia	MSCs were differentiated into more osteocytes and fewer adipocytes than 2D MSCs	Not reported	[44]
Adipose tissue	Hollow fiber bioreactor	Cryopreservation	Cryopreservation maintains capability differentiation	Freeze‒thaw does not interfere with AD-MSCs functional characteristics	[55]
Bone marrow	Wave-motion bioreactor	Pulsed electromagnetic fields	Enhancement of osteogenic transcription factors	Induce ROS-scavenging properties	[51]
Placenta	Spinner flask	3D spheroids	Trilineage differentiation potential comparable to 2D culture	Enhances immunosuppressive phenotype	[54]
Bone marrow (clonal line)	Perfusion bioreactor	Pulsed electromagnetic fields	Osteogenic differentiation increases with PEMF stimulation	PEMF induces the immune potential and promote angiogenesis	[50]
Amnion	Spinner flask	Not reported	Adipogenesis and osteogenesis differentiation are facilitated	Improve the inflammatory suppression	[56]
Warthon’s Jelly	Multilayer flask and stirred bioreactor	Not reported	Maintains trilineage differentiation potential	3D culture reduces CD105 due to the harvesting procedure without impact on multipotency	[10]
Warthon’s Jelly	Spinner flask	Dissolvable microcarriers	Multilineage differentiation potential is not affected	Dual production of MSC (WJ) and their derived EV is possible	[53]
Adipose tissue	Spinner flask	Agitation	Maintains trilineage differentiation potential	Intermittent stirring in the initial 24 h of cultivation potentiated cell adhesion. Cell growth was higher under continuous agitation.	[47]
Umbilical cord	Stirred tank bioreactor	Agitation	As the stirring speed increased, the stemness of cells decreased, and spontaneous differentiation occurred	Add stirring speed reduces the size of microcarrier aggregates but also inhibit cell proliferation	[49]

### Impact of Large-Scale Expansion on Immunosuppressive Properties

MSCs modulate immune responses, making them promising candidates for cell therapy and tissue engineering. Moreover, these cells are not inherently immunogenic. These characteristics provide MSCs with the capacity to avoid recognition by allogeneic T or natural killer cells [57]. MSCs subsequently express low levels of major histocompatibility complex (MHC) class II molecules and intermediate levels of MHC class I molecules and do not express costimulatory molecules such as B7-1, B7-2, CD40, or CD40 ligands [58]. Additionally, MSCs have immunosuppressive properties and can inhibit in vitro T-cell proliferation and the function of both naïve and memory T cells [59]. For example, UC-MSCs significantly inhibited the proliferation of both CD3 + CD4 + T helper cells and CD3 + CD8 + cytotoxic cells when cocultured with activated peripheral blood mononuclear cells [38].

Large-scale MSC production, which is required to generate sufficient clinical-grade cells, is offset by a reduction in their immunomodulatory capability, which typically occurs after long-term culture [35]. The findings summarized in Table 2 highlight the critical influence of culture conditions on the immunomodulatory functions of MSCs, particularly in large-scale expansion. Repeated passaging, as shown with UC-MSCs, significantly reduces their ability to suppress Th1 and Th17 responses and impairs Treg upregulation, likely due to the diminished secretion of growth factors, adhesion molecules, and anti-inflammatory mediators [60]. Similarly, BM-MSCs exhibit downregulation of hematopoietic supportive genes and secretome alterations following expansion [61, 62], suggesting a functional decline with in vitro aging. Moreover, the cell source itself modulates immune effects, with Wharton’s Jelly MSCs demonstrating superior PBMC suppression and greater secretion of immunosuppressive mediators than other sources [63]. Preconditioning strategies, such as hypoxia, inflammatory or hormonal priming, and apoptosis induction, can partially restore or enhance immunomodulatory potency by altering cytokine profiles and promoting Treg and Th2 responses [39, 64–66]. These results underscore the necessity of optimizing culture parameters and considering MSC sources in the design of large-scale production protocols to preserve the therapeutic efficacy of MSCs, particularly for their immunomodulatory applications.

**Table 2:** Effect of culture conditions on the Immunomodulatory properties of MSCs [39, 60–66]

Cell source	Factor	Effect on Immune system cells	Effects on the secretion profile	Ref.
Umbilical cord	Different passages	After passage 8, Th1 suppression and Th17 either upregulation of T reg is not possible	Passaging reduces the secretion of grow factors, cell adhesion and anti-inflammatory factors	[60]
Bone marrow	Primary or expanded cells	Not reported	Hematopoietic supportive genes are reduced on expanded cells	[61]
Bone marrow	Culture expansion, MSC source and culture medium	Not reported	Secretome and immunomodulatory potential are altered	[62]
Umbilical cord	Hypoxia	Not reported	The secretion of inflammatory factors is inhibited	[39]
Infrapatellar fat pad	Growth media and inflammatory/hormonal priming	XFSF attenuated PBMC proliferation. Promote M2 polarization	Not reported	[64]
Adipose tissue	Cell-free MSC extract	Priming extract suppresses lymphocyte activation and enhances T-cell expansion	Priming extract upregulated COX-2, TSG-6, and TGF- β1 molecules	[65]
Bone marrow, adipose tissue, Wharton’s Jelly, and decidua tissue	Cell source	WJ strongest inhibit PBMC proliferation	WJ has the highest secretory profile of PGE-2 and anti-inflammatory cytokines	[63]
Bone marrow and Wharton’s Jelly	Hypoxia and induction of apoptosis	Precondition induces Tregs cells, enhanced Th2 responses and mitigated Th1 and Th17 responses	Not reported	[66]

The immunomodulatory behavior of MSCs has been evaluated in preclinical and clinical studies. The principal mechanisms underlying the immunomodulatory capabilities of MSCs include cell signaling and paracrine activity. However, it remains difficult to understand the mechanism by which the variability of MSCs impacts their immunomodulatory capability. The administration of MSCs likely influences immunomodulatory mechanisms through their secretomes. Understanding the effects of chronic inflammatory cytokines and other factors on MSC-induced immunomodulatory effects could help identify new preconditioning approaches to promote the therapeutic efficacy of MSCs and weaken the variation in their paracrine ability for clinical applications [67].

### Quality Control Measures in Clinical-Scale MSC Production

In addition to MSC expansion, the scaling process for clinical applications must provide adequate therapeutic quality and purity of cells, according to GMP standards. Quality control testing usually includes viability, immunophenotyping, sterility, mycoplasma testing, and endotoxin level assessment, all of which can be performed using various assays [68]. Additionally, regular monitoring of key cellular markers and gene expression related to proliferation and senescence can serve as quality control measures to ensure the functionality of MSCs during large-scale culture [15]. However, during ex vivo expansion at a clinical scale, the biological functions of MSCs can be compromised by replicative senescence, making it difficult to harvest abundant high-quality clinical-grade cells. When robust differentiation into certain lineages and long-term expansion are necessary, exposure to physical, chemical, or biological cues can be used to prime MSCs toward specific cell types during differentiation. L-ascorbic acid-2-phosphate is a common supplement in chondrogenic induction media but is not commonly used for MSC expansion. It has been shown to have a beneficial effect on chondrogenic differentiation and to reduce the number of senescent cells during long-term expansion [69]. Additionally, to selectively reduce senescence-associated characteristics while preserving the differentiation capacity of expanded MSCs, the use of senolytic agents, such as fisetin, is a promising strategy for increasing the efficacy of MSC-based regenerative therapies [70]. A novel strategy for rejuvenating senescent MSCs without genetic manipulation or drug administration involves the use of electrical signals through a piezoelectric b-poly(vinylidene fluoride) film-based culture plate, which prevents MSC senescence by improving mitochondrial function and mitigating oxidative and glycoxidative stress [71]. However, ex vivo expansion of MSCs at higher passage numbers limits their clinical application because of senescence and variations in genetic stability. Thus, novel protocols involving repetitive culturing have been proposed for rapid MSC expansion without negatively affecting their characteristics [72]. Additionally, prolonged culture expansion to obtain sufficient cell doses from a single donor affects the final product quality and introduces heterogeneity in the clinical outcomes. Thus, pooling MSCs from multiple bone marrow donors has potential advantages in clinical applications. This approach reduces the heterogeneity of individual donors, achieves more stable proliferation, improves immunosuppressive capabilities, and reduces the number of senescent cells without compromising MSC properties [73].

Despite the importance of senescence in the therapeutic potential of MSCs, the cellular mechanisms and pathways involved in MSC senescence remain unclear. A recent study revealed that the miR-34a-HK1 signaling axis alleviates MSC senescence by enhancing glycolytic metabolism. This information provides a novel mechanism for delaying MSC senescence. The identification of biomarkers for MSC senescence could be promising for maintaining high-quality cells in the future. For example, the podoplanin glycoprotein strongly influences both stemness and senescence properties of MSCs, suggesting that it is a potential biomarker for enhancing the clinical utility of MSCs in regenerative medicine [74]. Another method for monitoring biological changes during MSC expansion is surface-enhanced Raman spectroscopy (SERS), which is cost-effective and efficient for rapid and precise quality control of MSC expansion [75]. Additionally, dual-drug-encapsulated liposomal nanoparticles (LNPs) can suppress senescence in AD-MSCs by reactivating mitophagy. Although this approach is highly promising for advancing cell therapies, the mechanism of action and optimal conditions for LNP treatment must be elucidated [76].

### In vivo and Clinical Implications

#### Translating In Vitro Findings to In Vivo Applications

*The in vitro* conditions during MSC culture are clear determinants of therapeutic efficacy. The transition from in vitro findings to in vivo applications has shown that the cellular microenvironment shapes the secretory profile, differentiation potential, and immunomodulatory potency of MSCs.

Hypoxic culture, which mimics the native physiological niche of MSCs (1–1% O2), has shown promising results in enhancing MSC function. As shown in a murine model of osteoarthritis, UC-MSCs expanded under long-term hypoxia retained genomic stability, had increased proliferative capacity, reduced senescence, and demonstrated superior anti-inflammatory efficacy [39]. Additionally, hypoxia-conditioned amniotic MSCs improve antimicrobial properties by increasing LL-37 secretion and accelerating wound healing in diabetic mice [77]. Another option for providing a biomimetic environment that closely resembles the in vivo tissue architecture for MSC enhancement is the use of 3D culture systems. When MSCs are cultured as gellan gum-based spheroids, they exhibit increased stemness and immunomodulatory gene expression [43].

In addition to physical modulation, pharmacological preconditioning and immune microenvironmental priming modify MSC functions in vivo. For example, metformin induces M2 macrophage polarization, creating an anti-inflammatory mechanism that enhances the osteogenic differentiation of BM-MSCs both in vitro and in vivo [78]. Moreover, retinoic acid preconditioning of MSCs promoted hyaluronic acid secretion and activated the CD44-PI3K/AKT signaling pathway in renal epithelial cells, which improved recovery in a mouse model of acute kidney injury [79].

In addition, biomaterials, such as gold nanoparticle-functionalized hybrid hydrogels, can improve the clinical properties of MSCs. In a rat sciatic nerve injury model, hydrogels protected MSCs from oxidative stress and improved neurotrophic factor secretion, leading to improved nerve regeneration [80]. In fact, the in vitro environment, such as oxygen conditions, culture models, priming, and the use of biomaterials, influences the clinical efficacy of MSCs.

Even with the outcomes of MSC therapy observed in animal models, translation to clinical practice requires an understanding of MSC behavior within a chronic inflammatory milieu and enhancement of MSC persistence and migratory capabilities in the body [81].

#### Clinical Studies and Trials

**Fig. 2:**
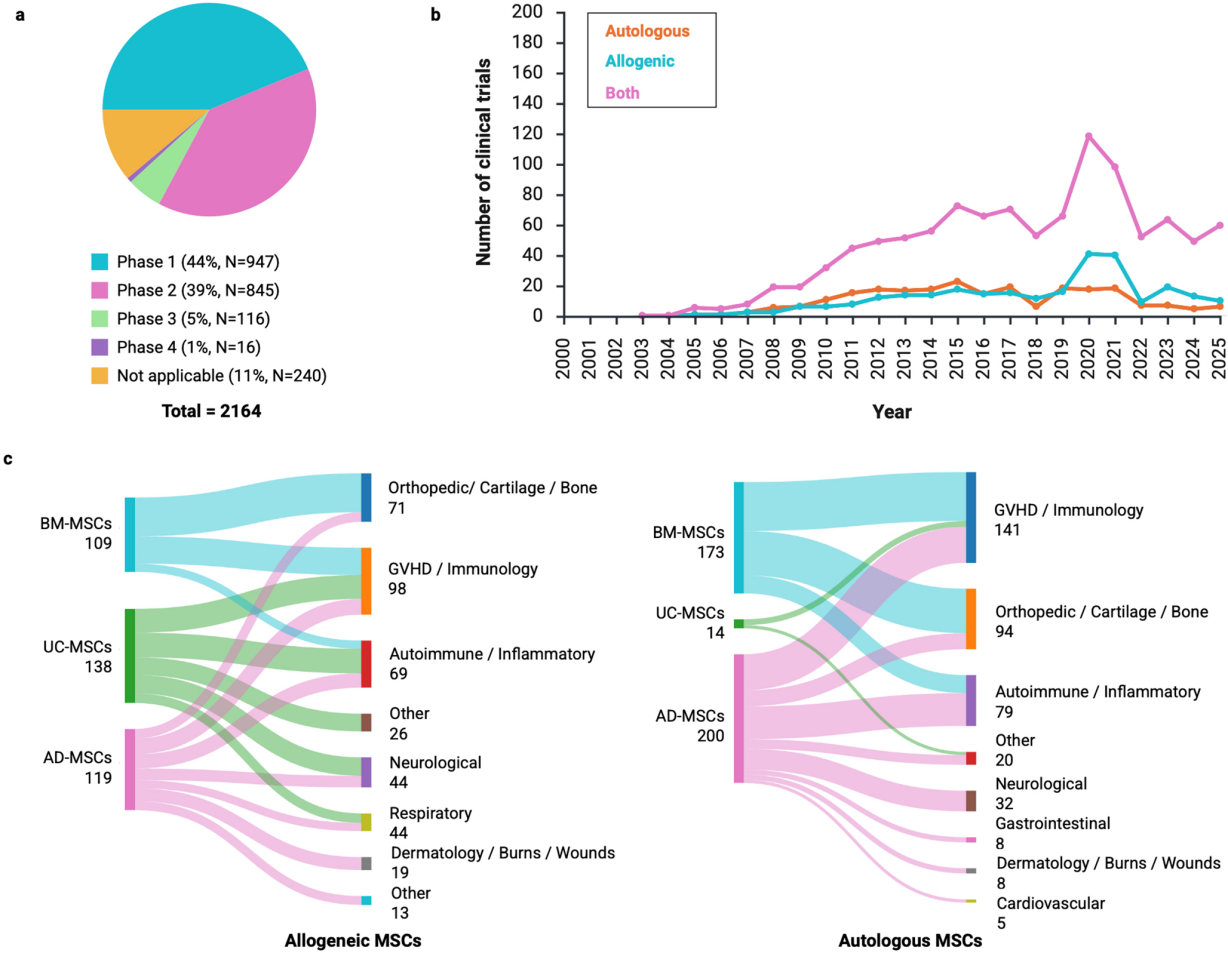
MSC-based cell therapies are listed at clinicaltrials.gov. (**A**) Number of trials in different phases of clinical research (where mesenchymal stem cells were used as a search term). (**B**) Number of autologous, allogeneic, and both types of cells listed over time. Among the total number of clinical trials listed, 733 failed to report the source of cells (where mesenchymal stem cells AND allogeneic or autologous cells were used as search terms). (**C**) Autologous and allogeneic MSC applications from different tissue sources (where mesenchymal stem cells AND allogeneic or autologous cells were used as search terms). (Created with BioRender.com and SankeyMATIC.)

By the beginning of September 2025, up to 2100 clinical trials involving human MSCs had been conducted, of which 83% were in phases I and II and only 6% were in phases III and IV (Fig. 2a). These clinical trials are either performed with patients’ own cells (autologous therapies) or with cells provided by healthy donors (allogeneic therapies). Autologous applications have been studied more than allogeneic applications, and the number of clinical trials has increased rapidly (Fig. 2b). MSCs are considered a treatment for a range of conditions in different areas, including orthopedics, immunology, neurology, dermatology, gastroenterology, and cardiovascular systems (Fig. 2c), owing to their potential to differentiate into multiple cell types, their paracrine activity, and their immunomodulatory properties.

Despite several clinical trials on the use of MSCs in treating human diseases, there are critical steps in translating MSCs into standardized therapeutics. These challenges include the inherent heterogeneity of MSC populations, which is influenced by the tissue source, donor characteristics, and culture conditions, creating significant batch-to-batch variability [82]. Therefore, for the development of standard-based MSC therapies, it is necessary to identify and evaluate the most reliable methods for MSC isolation, optimize cell culture systems, and derive sufficient high-quality MSCs [5]. Additionally, addressing issues such as MSC behavior in inflammatory environments and optimizing administration protocols are crucial for successful implementation in therapy [81].

### Future Directions and Research Gaps

#### Emerging Techniques and Innovations

As discussed, the heterogeneity of the cell population is a challenge for therapeutic efficacy without standardized protocols for MSCs. Therefore, new and innovative alternatives have been developed for these methods. To ensure a homogeneous population, different cell types can be separated after cell differentiation using the dielectrophoretic separation technique. This is due to dielectrophoretic changes in MSCs when they begin differentiating. These modifications enable cell separation during the early stages of differentiation [83].

Current clinical guidelines recommend limiting MSC expansion to early passages because of the progressive loss of function associated with replicative senescence. Strategies that selectively eliminate senescent cells during expansion have the potential to yield clinically effective MSC populations. For example, microfluidic platforms have been employed to exploit differences in cell migration dynamics to distinguish senescent MSCs from presenescent MSCs. Cells exhibiting greater migratory capacity display reduced DNA damage and lower levels of senescence markers thereby allowing the selection of more functionally potent subpopulations [84]. Enhancing the homogeneity of the administered MSCs through such separation may ultimately improve long-term therapeutic outcomes. Furthermore, deep learning models, such as ResNeXt architectures trained to recognize the features of natural cellular aging, have demonstrated utility in predicting the passage number and senescence status of MSCs, offering a powerful tool for quality control in clinical manufacturing [85].

#### Recommendations for Future Research

As described above technical limitations are encountered during the large-scale expansion of MSCs. To guide future efforts in MSCs manufacturing, it is essential to systematically identify and address the technical bottlenecks that limit the scalability and therapeutic consistency of MSCs. Table 3 summarizes the main limitations of MSCs and presents current strategies and emerging techniques to reduce their negative effects.

**Table 3:** Key limitations and mitigation strategies in Large-Scale MSC expansion [9, 15, 37, 39, 44, 46–48, 54, 63, 69, 70, 73, 83, 86]

Limitation	Description	Mitigation strategies	References
Replicative senescence	Progressive loss of proliferation and multipotency with increasing passage numbers.	Antioxidants (ascorbic acid), senolytic agents (e.g., fisetin), hypoxic conditions, and early passage use.	[39, 46, 69, 70].
Cellular heterogeneity	Donor-to-donor variability and batch inconsistency reduce the reproducibility.	Pooling MSCs from multiple donors; dielectrophoretic separation technique	[73, 83]
Bioprocess-induced mechanical stress	High shear forces in bioreactors impair MSC viability and their phenotype.	Low-shear bioreactor designs, optimized microcarrier systems, and intermittent agitation protocols.	[16, 47, 48]
Oxygen and nutrient gradients	Large-scale systems introduce heterogeneous microenvironments that affects cell behavior.	Perfusion systems with controlled oxygenation and nutrient delivery and in situ sensors.	[15, 44, 54]
Functional decline	Diminished differentiation and immunosuppressive function after prolonged culture.	Priming with biological/chemical cues; potency assays assessing immune and differentiation functions.	[69, 70]
Regulatory and standardization challenges	Lack of harmonized potency assays and release criteria across GMP facilities.	Development of advanced functional assays (e.g., secretome, omics) and regulatory harmonization efforts.	[9, 37, 44, 63]

Replicative senescence, one of the most prominent challenges, leads to reduced proliferative capacity and multipotency, particularly at higher passages. This is accomplished through the use of antioxidant supplements (e.g., ascorbic acid) [69] and senolytic agents (e.g., fisetin) [70], and optimizing expansion conditions, such as hypoxia [39] and early passage harvesting [46].

Additionally, donor variability and functional drift can be mitigated by pooling MSCs from multiple donors [73], performing cell differentiation via the dielectrophoretic separation technique [83], and using priming strategies to increase their immunomodulatory and differentiation potential [39, 64–66].

Other bioprocess-related stresses, including mechanical shear and nutrient gradients in bioreactors, compromise cell viability and functions [7]. Strategies such as intermittent-shear bioreactor designs, perfusion systems with controlled environmental parameters [47, 48], and microcarrier optimization have been implemented to alleviate these stresses [16].

Moreover, oxygen and nutrient gradients in large culture systems generate non-uniform microenvironments, leading to inconsistent cell behavior. This has been mitigated by employing perfusion systems with dynamic oxygenation and nutrient delivery, supplemented by real-time, in situ monitoring [15, 44, 54].

A key biological consequence of prolonged culture is functional decline, which manifest as reduced immunomodulatory and differentiation capabilities. Functional priming via bioactive molecules, and rigorous potency assays, are critical for ensuring therapeutic efficacy [69, 70].

Finally, regulatory and standardization challenges persist owing to inconsistent potency assay implementation and a lack of harmonized release criteria. Ongoing efforts to integrate omics-based profiling, secretome analysis, and advanced functional assays aim to align GMP practices across facilities and accelerate regulatory approvals [9, 37, 44, 63].

## Limitations

This article is a narrative review and, therefore, does not provide a formal systematic assessment of study quality or a quantitative synthesis of effect sizes. The available evidence is also highly heterogeneous across MSC tissue sources, donor characteristics, culture media, passage/PDL ranges, and scale-up platforms (planar systems, microcarriers, and distinct bioreactor configurations), which limits direct cross-study comparability and precludes the establishment of universal thresholds for potency loss. In addition, publication bias and inconsistent reporting of critical process parameters (e.g., oxygen tension, shear exposure, feeding strategy, and harvest methodology) may have influenced the apparent magnitude and direction of the observed effects. Finally, many potency and quality control assays remain context-dependent, and their predictive value for clinical outcomes is still evolving; thus, the translational implications discussed here should be interpreted as evidence-informed guidance rather than prescriptive standards.

## Conclusion

Large-scale MSC expansion is indispensable for clinical translation yet it imposes a persistent scalability-functionality trade-off driven by replicative senescence, bioprocess stress, and donor-dependent heterogeneity. Next-generation biomanufacturing should prioritize integrated mitigation strategies that combine well-controlled culture platforms with evidence-based passage/PDL limits and robust, multiparametric quality control. In particular, potency assays aligned with immunomodulation and secretome-related function, complemented by emerging single-cell and omics-enabled analytics, are likely to improve batch consistency and clinical relevance. Ultimately, tighter alignment between mechanistic understanding, scalable engineering, and regulatory guidance (including acceptance criteria for senescence thresholds, PDL limits, and allowable heterogeneity) will be pivotal in delivering safe, reproducible, and clinically effective MSC-based therapies.

## Data Availability

No datasets were generated or analyzed during the current study.
